# Cell-free DNA from human plasma and serum differs in content of telomeric sequences and its ability to promote immune response

**DOI:** 10.1038/s41598-017-02905-8

**Published:** 2017-06-01

**Authors:** Alzbeta Zinkova, Iva Brynychova, Alexander Svacina, Marie Jirkovska, Marie Korabecna

**Affiliations:** 1 Department of Biology and Medical Genetics, First Faculty of Medicine, Charles University and General Faculty Hospital in Prague, Albertov 4, 128 00 Prague, Czech Republic; 2 Department of Anthropology and Human Genetics, Faculty of Science, Charles University, Vinicna 7, 128 43 Prague, Czech Republic; 3 Department of Histology and Embryology, First Faculty of Medicine, Charles University, Albertov 4, 128 00 Prague, Czech Republic

## Abstract

Circulating cell-free DNA (cfDNA) may be involved in immune response regulation. We studied the variations in abundance of telomeric sequences in plasma and serum in young healthy volunteers and the ability of cfDNA contained in these samples to co-activate the TNF-α m RNA expression in monocytes. We performed qPCR to determine relative telomere length (T/S ratios) in plasma, serum and whole blood of 36 volunteers. Using paired samples of plasma and serum and DNase treatment, we analysed the contribution of cfDNA to the co-activation of TNF-α mRNA expression in THP1 monocytic cell line. We found significant differences between paired plasma and serum samples in relative T/S ratios (median 1.38 ± 1.1 vs. 0.86 ± 0.25, respectively) and in total amounts of cfDNA and in estimated total amounts of telomeres which were significantly higher in serum than in plasma. TNF-α mRNA expression in THP1 cells increased significantly after DNase treatment of all samples used for stimulation. The highest TNF-α mRNA expressions were observed after stimulation with DNase treated serum samples. Our results suggest that the different content of telomeric sequences in plasma and serum may contribute to the tuning of immune response. Further studies of this interesting phenomenon are needed.

## Introduction

Telomeres are known to prevent deleterious shortening of linear DNA of eukaryotic chromosomes during DNA replication. Telomere DNA contains G-rich repetitive sequences (TTAGGG in mammals). Telomeres progressively shorten after each cell division. They can be extended by the enzyme telomerase which is active mainly in embryonic stem cells^1^. The telomeres shorten not only with replication but also as the consequence of oxidative stress^2^. If the length of telomere sequences reaches a critical minimum, the telomeres are not more able to fulfil their tasks and the state of such cells may be described as replicative senescence. Therefore the length of telomeres might be used as an indicator of cellular aging and predictor of mortality. The shorter leukocyte telomere length (LTL) is reported to be associated with older age, male sex, Caucasian race, and possibly atherosclerosis but there is controversy with regard to association of LTL with other diseases of aging. Therefore the crucial question of whether shortening of the telomeres is a cause or a symptom of aging remains unanswered^3^. The length of telomere sequences is studied under wide clinical conditions with the goal to find markers distinguishing different health statuses of patients and allowing prognosis of their clinical outcomes^4, 5^. The comparison of different studies is difficult because different methodological approaches are used on various types of biological samples^3^. The attempt to compare and standardize the methodologies was already made^6^. The application of qPCR allows the estimation of the abundance of telomeric sequences in comparison with a single copy gene sequence.

Cell-free DNA (cfDNA) is recently studied with regard to its potential use as the source of useful biomarkers in various fields of medicine. The examination of foetal fraction of cfDNA in maternal circulation has been elaborated for non-invasive screening for foetal aneuploidies and chromosomal aberrations^7^. After establishment of methodologic approaches based on Next Generation Sequencing, the examination of cfDNA circulating in plasma became to be widely used for tumour detection and follow-up in oncologic patients^8, 9^. The elevated levels of cfDNA or the detection of specific gene sequences in its pool in circulation are reported in numerous clinical conditions associated with inflammation, tissue damage and cancer^10–13^. The detection of donor sequences in the cfDNA pool represents the important tool for monitoring of transplanted patients^14^. Levels of total cfDNA increase also after intensive physical activity^15, 16^.

Despite all above mentioned applications, the origin of cfDNA in circulation and the mode of its clearance are not fully understood. It is known that serum samples contain more cfDNA than the paired plasma samples^17^, examination of serum may be used to enhance the detection of cancer specific mutations in cfDNA^18^. The phenomenon of NETosis (Neutrophil Extracellular Trap) as quite recently described type of cell death^19^ could potentially provide an explanation of the different concentrations of cfDNA in paired plasma and serum samples as well as at least in some clinical situations associated with inflammation in which cfDNA levels in circulation are increased^20^. The main feature of NETosis is the formation of the so called extracellular traps which contain genomic DNA and wide spectrum of proteins including citrullinated histones and the enzymes released from neutrophil granules. The primary purpose of these nets formed preferentially by activated neutrophils is the entrapment of invading bacteria in circulation but NETosis may be stimulated by various signals also under sterile conditions and not only in neutrophils^21^. Platelet activation belongs to the well-known activators of NETosis^21, 22^.

Thrombi form on all known biomaterials partly due to platelet-mediated reactions and partly due to coagulation of blood plasma itself (the contact activates the blood zymogen FXII (Hageman factor) into an active enzyme form FXIIa^23^. Fuchs *et al*.^24^ found that NETs are liberated during storage of non-leukoreduced red blood cells (RBC).

Despite all these facts, the biological roles of cfDNA in circulation are very rarely studied^25^. In this study, we compared the relative amount of telomeric sequences in paired samples of plasma and serum in young healthy volunteers. We examined the effects of selected paired samples of plasma and serum on the stimulation of TNF-α (Tumor Necrosis Factor-α) mRNA expression in monocytic cell line THP1 before and after DNase treatment of these biological fluids.

To our best knowledge, we present the first study demonstrating the different effects of plasma and serum of young healthy individuals on the regulation of immune response.

It seems that especially telomere sequences in the pool of cell-free DNA in circulation may play regulatory roles with regard to the fine tuning of immune system. Vertebrate DNA modified by apoptosis stimulates Toll-like receptor 9 (TLR9) which activates nuclear transcription factors including NFκB. These transcription factors promote increased expression and release of wide spectrum of immune-regulatory molecules^26^ but telomeric sequences are able to block TLR9^27, 28^. Therefore we focused also on analysis of content of telomeric sequences in plasma and serum samples together with examination of their stimulatory effects on THP1 cells. Relative content of telomeric sequences in whole blood samples was examined due to the known fact that blood cells represent the main source of cfDNA in circulation^29^.

## Results

### Relative quantification of telomeric sequences

The construction of standard curves and the principles of calculations (total amount of cfDNA and T/S ratio) are described in Methods. The standard curves were set at the start of experiments and used for the entire study. Due to nonparametric data distribution, we report medians and standard deviations in the following text.

**Figure 1:**
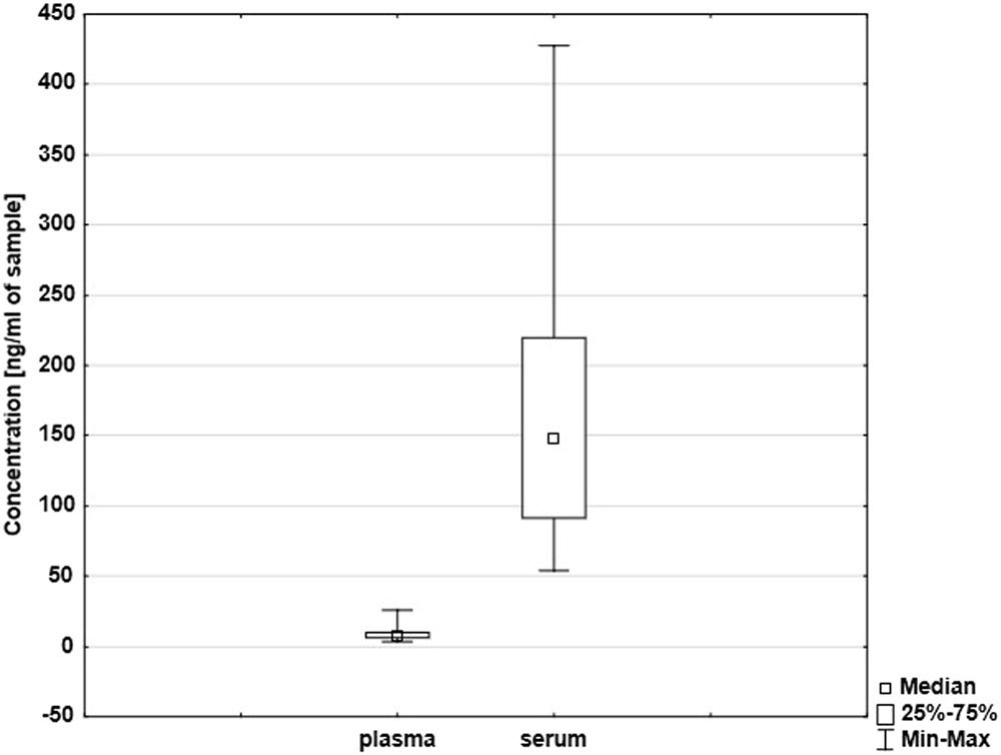
Box plot showing different concentrations of total cfDNA in plasma and serum samples [ng/ml] measured by qPCR for single copy gene *36B4* (n = 36).

The total amount of cfDNA measured using qPCR on single copy gene was significantly (p < 0.0001, Wilcoxon test) higher in serum (148.13 ± 91.31 ng/ml, range 54.33–427.30 ng/ml) than in plasma (7.48 ± 5 ng/ml, range 3.16–26.05 ng/ml). Box plots are shown in Fig. 1. No correlations in total amounts of cfDNA between plasma and serum samples were detected in paired samples.

**Figure 2:**
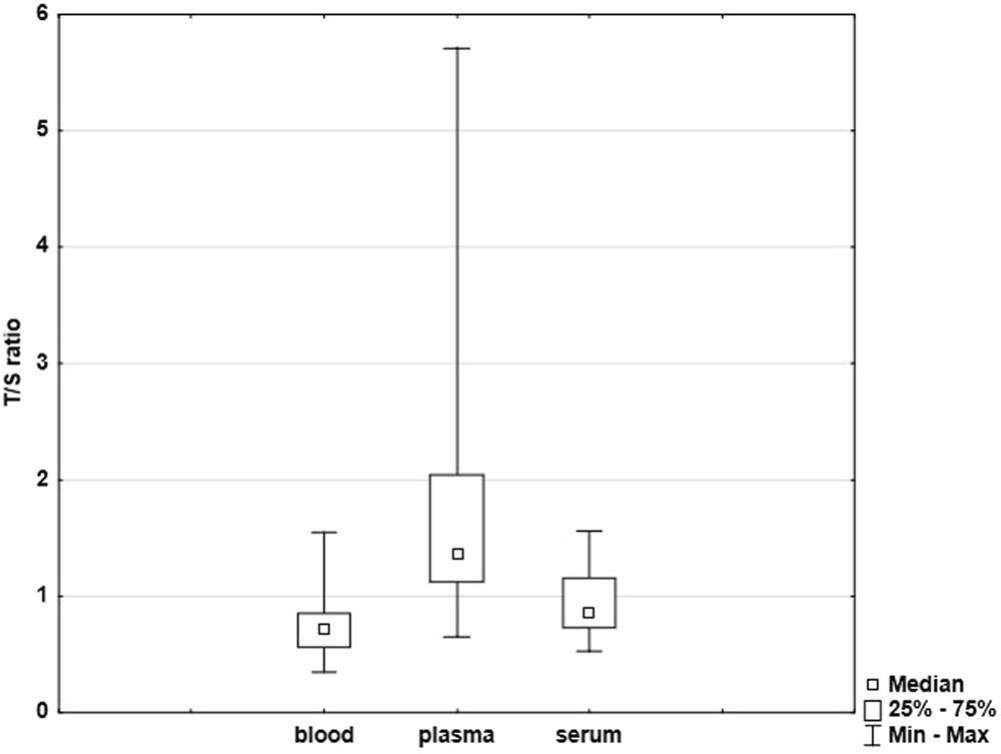
Box plot showing relative T/S ratios for whole blood, plasma and serum samples (n = 36).

**Figure 3:**
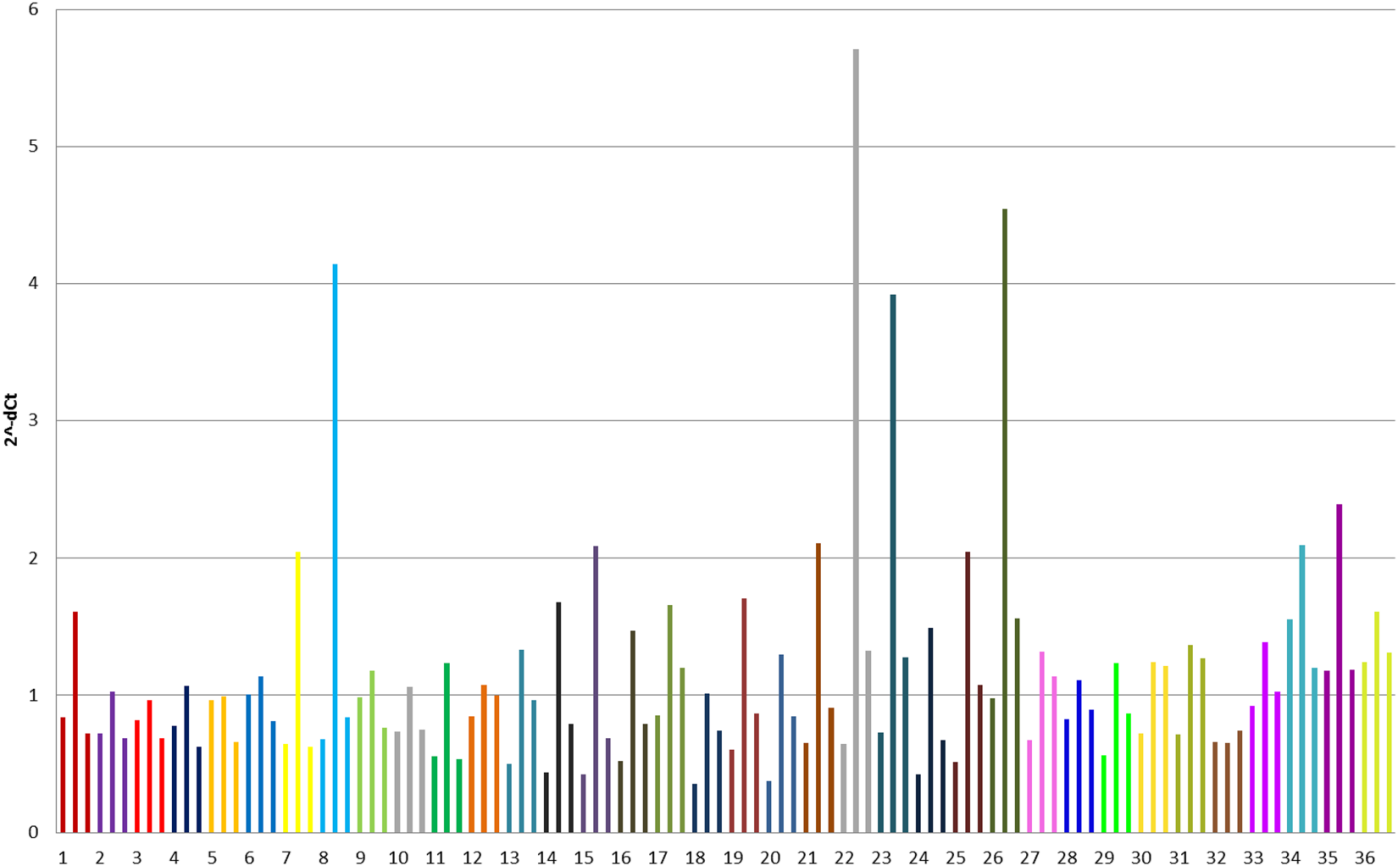
Differences in relative T/S ratios among whole blood, plasma and serum samples in 36 healthy young volunteers. Individuals are marked by different colours. The order of samples for each individual is following: whole blood, plasma and serum.

Significant differences in the relative T/S ratios were found among whole blood, plasma and serum samples. These ratios were significantly (p < 0.0001, Dunn test) higher in plasma than in serum samples (1.38 ± 1.1 vs. 0.86 ± 0.25, respectively) and in plasma than in whole blood samples (1.38 ± 1.1 vs. 0.72 ± 0.25, respectively). In serum samples, we observed higher ratios than in whole blood samples too (0.86 ± 0.25 vs. 0.72 ± 0.25, respectively, p = 0.0225, Dunn test). All these results are documented in Figs 2 and 3.

Figure 3 clearly demonstrates that the relative T/S ratios in all types of biological material in nearly all examined subjects follow the similar scenario with the highest values in plasma, intermediate values in serum and the lowest ones in whole blood.

### Stimulations with paired plasma and serum samples with known T/S ratios and cfDNA concentrations

For stimulations, we selected randomly paired plasma and serum samples of individuals no. 12, 13, 16, 18, 21, 22, 27, 31, 33 and 36 (Fig. 3). Relative T/S ratios from previous experiments were reanalysed for this chosen samples and significant differences in the relative T/S ratios were confirmed again (plasma 1.38 ± 1.32 vs. serum 1.01 ± 0.2, p = 0.005, Wilcoxon test) (Table 1). Plasma and serum samples treated by DNase were examined by qPCR for single copy gene (*36B4*) and they do not revealed any amplification signals.

**Table 1:** Selected characteristics of samples used for stimulatory experiments. Wilcoxon signed-rank test was used.

Individual. No.	T/S ratio		ETAT [ng/ml]	
	Plasma	Serum	Plasma	Serum
12	1.08	1.00	10.15	163.24
13	1.33	0.96	34.29	73.99
16	1.48	0.80	10.98	85.64
18	1.01	0.75	7.69	69.78
21	2.11	0.9	16.51	248.43
22	5.71	1.33	47.79	150.37
27	1.32	1.14	8.01	357.47
31	1.37	1.27	15.17	307.79
33	1.39	1.03	10.37	71.04
36	1.61	1.31	25.38	188.26
p values	0.005		0.005	

**Figure 4:**
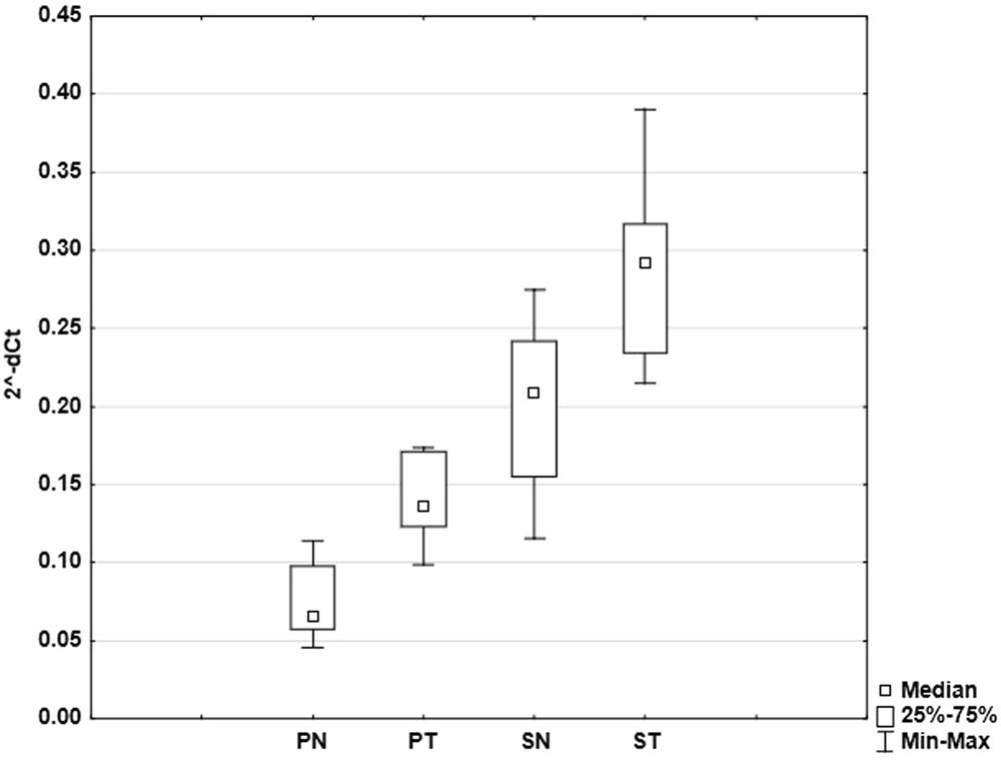
Graph showing the relative TNF-α mRNA expression after stimulation of THP1 cells with different types of sample (PN – non-treated plasma, PT – plasma treated with DNase, SN – non-treated serum, ST – serum treated with DNase). Bars show standard deviations (n = 10).

In case of stimulation by DNase treated and non-treated plasma, we found significant difference (p = 0.005) between the levels of TNF-α mRNA expression. DNase treatment of plasma samples led to statistically significant enhancement of their stimulatory activity in the term of increase of relative TNF-α mRNA expression (0.14 ± 0.02 vs. 0.07 ± 0.02, respectively) (Fig. 4). The same trend was observed between treated and non-treated serum samples (0.29 ± 0.05 vs. 0.21 ± 0.05 respectively, p = 0.013). Statistically significant (p = 0.005, Wilcoxon test) differences were observed in stimulation activity between non-treated plasma and serum samples. DNase non-treated serum samples stimulated the relative TNF-α mRNA expression in higher extent than non-treated plasma samples (0.21 ± 0.05 vs. 0.07 ± 0.02, respectively). DNase treated serum samples had also higher stimulatory effect than treated plasma samples T (0.29 ± 0.050 vs.14 ± 0.02, respectively). The results of stimulatory experiments are summarized in Fig. 4.

When we compared the performance of all ten quartets of samples in stimulatory experiments we observed again nearly the identical scenario in all examined individuals: The lowest values of relative TNF-α mRNA expression were found when the cells were stimulated using DNase non-treated plasma samples. After DNase treatment, the plasma samples enhanced their stimulatory capacity (fold change difference 1.86) which was still lower or almost identical as stimulatory capacity of DNase non-treated serum samples. DNase treatment of serum samples in most cases enhanced their stimulatory capacity (fold change difference 1.4) above the capacity of non-treated serum.

To estimate the content of telomeric sequences in each sample used for stimulatory experiment, we calculated ETAT values as described in Methods. We found significant differences (p = 0.005, Wilcoxon test) between ETAT values of plasma and serum samples (Table 1).

## Discussion

In agreement with previously known facts^17^, we found statistically significant differences between the total amounts of cfDNA in plasma and serum with considerably higher levels in serum. Such results consistently found in most of individuals examined in this study provided the evidence that there were no pre-analytical events leading to more intensive degradation of cfDNA in serum samples.

Our comparison of relative T/S ratios between plasma and serum with significantly higher values in plasma demonstrates probably the different mode of physiological processing of cfDNA in these biological fluids. Serum contains higher content of telomeric sequences than plasma (higher ETAT values in this study) due to its higher amounts of total cfDNA.

Serum formation itself is a very complex process dependent on coagulation mechanisms which are associated with the activation of platelets and release of pro-inflammatory cytokines^30^. This fact is in good agreement with the higher stimulatory activity of native serum samples in comparison with plasma samples in our experiments on THP1 monocytic cell line. NETosis activated by complex stimuli during coagulation and serum formation^21, 22^ may be the main source of elevated levels of total cfDNA in serum.

Our comparison of T/S ratios in whole blood, plasma and serum provides the first evidence for the existence of specific mechanisms which are responsible for maintenance of telomeric sequences in circulation. The origin of cfDNA could determine the character and alternatively the mode of clearance of specific sequences in their pool in circulation - cfDNA released during NETosis may be decorated with specific proteins - e.g. citrullinated histones^24^ and therefore easily recognizable by molecular mechanisms.

It seems that especially telomeric sequences in circulation may play regulatory roles in the fine tuning of immune system. Some studies^27, 28^ provide the evidence that the telomeric sequences are able to block TLR9 and to inhibit immune response and that this activity is associated with the ability of telomeric repeat sequences to form 3-dimesional structures based on quainine-quadruplexes^26^.

For example, it is experimentally proven that artificially prepared telomeric sequences are able to inhibit proliferation of B-cells regulated via TLR^28^.

With regard to the interpretation of our stimulation experiment, it is necessary to keep in mind that cfDNA and telomeric sequences contained in its pool are not the only regulators of immune response in such complex biological fluids. Our experiments clearly demonstrated the difference in the stimulatory properties of paired DNase non-treated samples of plasma and serum of identical individuals. Stimulatory effects of non-treated serum samples were higher than the effect of non-treated plasma (Fig. 4). The experiments with the complete removal of DNA using DNase digestion demonstrated clearly the important role of cfDNA and its telomeric sequences in plasma and serum of healthy young individuals in the inhibition of immune response. The results are in full agreement with the known facts about the involvement of cell-free telomeric sequences in the regulation of immune functions^26–28^.

The telomere sequences in plasma samples were examined till today only in two studies^31, 32^. Wu and Tanaka found significantly shortened telomeres in cfDNA in plasma of breast cancer patients with no prior treatment^32^. Idei *et al*. reported similar results in patients with ovarian cancer^31^. Two studies were focused also on analysis of telomere sequences in serum. Fu *et al*. conducted a study to determine the relative telomere length (RTL) in serum samples from hepatitis B virus (HBV) -related hepatocellular carcinoma patients and cancer-free HBV controls^33^. Cancer patients had a significantly longer RTL in cfDNA in serum. Wan *et al*. found significantly longer RTL in cfDNA in serum in cirrhotic cases than in non-cirrhotic controls and proposed the determination of RTL in serum as suitable non-invasive marker for detection of patients with the risk of cirrhosis^34^. The terms “longer” or”shorter” telomeres in the texts of these publications are inappropriate because the methodologies used by these researchers examine rather the abundance of telomeric sequences in samples and are not able to provide the results documenting the lengths of the longest and/or shortest telomeres in examined samples. The results of such studies analysing the abundance of telomeric sequences in plasma or serum in cancer patients should be interpreted in association with the selected markers describing the functions of immune system of examined individuals to allow better evaluation of observed changes in the context of immune response associated with tumour development.

Our results document the existence of molecular mechanisms which are responsible for maintenance of appropriate levels of free telomeric sequences in human circulation and the role of cfDNA in plasma and serum in the regulation of immune response. According to our results, it seems that telomeric sequences in native plasma and serum samples may contribute to the inhibition of TNF-α mRNA expression in monocytes. Further studies are needed to elucidate the character of these physiological regulatory events and the consequences of their disturbances for human health.

## Methods

### Subjects

Healthy volunteers (n = 36, 17 men and 19 women) – participants of the study – were 20 to 25 years old students. Blood withdraws were performed during the everyday student’s activities without any excessive physical load. The study was approved by the Ethical committee of the 1^st^ Faculty of Medicine of Charles University and General Faculty Hospital in Prague. Informed consent was obtained from all participants included in the study. All methods were performed in accordance with the relevant guidelines and regulations.

### Blood, serum and plasma sampling

This study compares content of telomere sequences in three types of biological samples – whole blood, plasma and serum. In case of plasma and whole blood, the blood was collected into tubes containing EDTA (BD Vacutainer), serum samples were collected into tubes with clot activator and polymer gel (BD Vacutainer). The whole blood (300 μl) for DNA isolation was taken from EDTA tubes before the processing of samples for plasma isolation. For plasma and serum isolation, the whole blood was centrifuged at 2600 rpm for 10 min at 10 °C. After this procedure, the collected material was centrifuged once again in 2 ml low-bind tubes at 14500 rpm for 10 min to remove residual cells. All samples were stored at −20 °C until DNA and cfDNA isolation.

### DNA isolation and qPCR

CfDNA from 1 ml plasma or serum was isolated by QIAamp® Circulating Nucleic Acid Kit (QIAGEN, Germany) according to the manufacturer’s instructions and eluted in 50 μl of elution buffer and 5 μl of this solution was used for each amplification reaction. Whole blood DNA was isolated by QIAamp® DNA Blood Mini Kit (QIAGEN, Germany) and eluted in 200 μl of elution buffer and then diluted 15-fold and also 5 μl of this diluted solution was used for each amplification reaction.

DNA isolations were immediately followed by qPCR which was performed at QuantStudio™ 12 K Flex Real-Time PCR System (Applied Biosystems, USA). Final volume of a PCR reaction was 25 μl, one half of this volume consisted of Power SYBR® Green PCR Master Mix (Life Technologies, USA). Primers for amplification of telomere sequences (“telomeres”) and single copy gene *36B4* (coding acidic ribosomal phosphoprotein PO) were chosen from literature^35, 36^. Final concentrations of primers were 300 nM for single copy gene (*36B4*) and 900 nM for telomeres (*tel*). The primer sequences were as follow: *36B4*u: 5′-CAG CAA GTG GGA AGG TGT AAT CC-3′; *36b4*d: 5′-CCC ATT CTA TCA TCA ACG GGT ACA A-3′^35, 36^; *tel 1 1b*: 5′-CGG TTT GTT TGG GTT TGG GTT TGG GTT TGG GTT TGG GTT-3′; *tel 1 2b*: 5′-GGC TTG CCT TAC CCT TAC CCT TAC CCT TAC CCT TAC CCT-3′^36^. All reactions were run in triplicates. The hold stage which activated the enzyme included 2 steps: 50 °C for 2 min and 95 °C for 2 min. This stage was followed by 40 cycles of PCR: 95 °C 30 s and 54 °C 1 min. At the end of each run, there was a dissociation step (default software setting). Ramp rates between all steps were 1.6 °C/s. The standard curves for both amplification reactions were constructed before the processing of samples. Standard DNA was diluted and used at concentration 5–0.312 ng per reaction. Amplification efficiency was 90.85% for *36B4* (slope −3.562, R^2^ 0.998 and error 0.041), and 97.94% for “telomeres” (slope −3.372, R^2^ 0.998 and error 0,043).

In each PCR run, the inter-run calibration reaction containing 20 ng of standard DNA was included. For construction of standard curves, for inter-run calibration and for calculation of relative T/S ratio, the standard DNA (Taq Man Control Genomic DNA, Applied Biosystems, USA) was used.

The T/S ratio was calculated as 2^−[Ct (telomeres)/Ct (36B4)]^ = 2^−∆Ct^. To determine the relative T/S ratio of the sample, its T/S ratio was normalized to ∆Ct of the standard. The final formula for relative T/S ratio was 2^−(∆CtSample−∆CtStandard)^.

The logarithm of the total amount of cfDNA (*x*) was calculated using the experimental data for *36B4* and following equation: *y* = *Ax* + *B* where *A* is slope, *B* is Y-inter, and *y* is Ct.

### Cell cultivation and stimulation

For stimulations, paired samples of plasma and serum from 10 young healthy volunteers were selected from the sample set analysed in previous experiments leading to the determination of T/S ratios and total cfDNA in these samples. At least 5 × 10^5^ cells of THP-1 monocytic cell line were cultivated in 24 well plate (Orange Scientific, Belgium) in 1 ml of RPMI 1640 medium (Sigma Aldrich, USA) supplemented with 10% plasma or serum of healthy volunteer, 200 mM L-glutamine and 1% penicillin/streptomycin (Sigma Aldrich, USA).

Each sample of plasma and serum was divided into two technical replicates. One of each replicate was treated by DNase Turbo DNA-free^TM^ (Ambion by Life Technologies, USA) and also added in 10% concentration to the cultivation medium and used in parallel with its non-treated replicate in the stimulatory experiment. In plasma samples, the DNases are inhibited by EDTA immediately after blood withdraws. EDTA chelates divalent ions which are essential for the activity of these enzymes^37^. The DNase Turbo kit contains a specific buffer for proper supplementations of reaction with ions to allow the DNase activity. All four samples (DNase – treated and non-treated plasma, DNase – treated and non-treated serum) from one healthy volunteer were stimulated at 37 °C in 5% CO_2_ for 3 hours. After this interval, samples were centrifuged at 14 000 g for 6 min. Supernatant was removed and cells were resuspended in 500 μl Lysis Solution (GenElute^TM^ Mammalian Total RNA Miniprep Kit, Sigma Aldrich, USA).

### RNA isolation, reverse transcription and qPCR

RNA was isolated by GenElute^TM^ Mammalian Total RNA Miniprep Kit (Sigma Aldrich, USA). Extracted RNA was reverse-transcribed into cDNA by HyperScript^TM^ One-step RT-PCR Master mix (GeneAll, Korea). QPCR was performed in LightCycler®480 System (Roche Applied, USA) with Assays-on-Demand TaqMan® Gene Expression Assay Mix (Life Technologies-Applied Biosystems,USA) for *PGK1* Hs99999906_m1 (Phosphoglycerate Kinase-1) as a reference gene and *TNF-α* Hs01113624_g1. The expressions of both genes were examined in all samples, the levels of TNF-α mRNA expression were given as = 2^−∆Ct^ related to the reference gene *PGK1*.

### Statistical analysis

We used nonparametric statistical tests due to small sample sizes. We employed Wilcoxon test (STATISTICA, StatSoft, USA) for paired samples and Friedman tests for multiple samples (comparison of whole blood, plasma and serum). We used significance level α = 0.05 for all comparisons.

### cfDNA analysis

We performed Friedman rank-sum at R environment to reveal any differences among whole blood, plasma and serum. Then we used Dunn post hoc test with Bonferroni correction to find out T/S ratio differences among blood-plasma, blood-serum and plasma-serum samples.

### Analysis of stimulation experiments

Wilcoxon signed-rank tests were used for all comparisons. To study the amount of telomeric sequences in samples used for stimulation, ETAT (Estimated Total Amount of Telomeres) was calculated as T/S ratio of the sample multiplied by its total cfDNA concentration determined by qPCR on single copy gene using calibration curve for this gene.
